# Genomic Diversity of *Listeria monocytogenes* Isolated from Clinical and Non-Clinical Samples in Chile

**DOI:** 10.3390/genes9080396

**Published:** 2018-08-02

**Authors:** Viviana Toledo, Henk C. den Bakker, Juan Carlos Hormazábal, Gerardo González-Rocha, Helia Bello-Toledo, Magaly Toro, Andrea I. Moreno-Switt

**Affiliations:** 1 Escuela de Medicina Veterinaria, Facultad de Ciencias de la Vida, Universidad Andres Bello, Santiago 8320000, Chile; v.toledoneira@uandresbello.edu; 2 Center for Food Safety and Department of Food Science and Technology, University of Georgia, Griffin, Athens, GA 30602, USA; hendrik.denbakker@uga.edu; 3 Departamento de Laboratorio Biomédico, Instituto de Salud Pública de Chile, Santiago 7750000, Chile; jchormazabal@ispch.cl; 4 Laboratorio de Investigación en Agentes Antibacterianos, Departamento de Microbiología, Facultad de Ciencias Biológicas, Universidad de Concepción, Concepción 4030000, Chile; ggonzal@udec.cl (G.G.-R.); hbello@udec.cl (H.B.-T.); 5 Millennium Nucleus on Interdisciplinary Approach to Antimicrobial Resistance, Las Condes 12496, Lo Barnechea, Santiago 7690000, Chile; 6 Instituto de Nutrición y Tecnología de los Alimentos (INTA), Universidad de Chile, Macul, Santiago 7810000, Chile; magaly.toro@inta.uchile.cl

**Keywords:** *Listeria monocytogenes*, whole genome sequencing, single nucleotide polymorphism, genomic diversity, Chile

## Abstract

*Listeria monocytogenes* is the causative agent of listeriosis, which is an uncommon but severe infection associated with high mortality rates in humans especially in high-risk groups. This bacterium survives a variety of stress conditions (e.g., high osmolality, low pH), which allows it to colonize different niches especially niches found in food processing environments. Additionally, a considerable heterogeneity in pathogenic potential has been observed in different strains. In this study, 38 isolates of *L. monocytogenes* collected in Chile from clinical samples (*n* = 22) and non-clinical samples (*n* = 16) were analyzed using whole genome sequencing (WGS) to determine their genomic diversity. A core genome Single Nucleotide Polymorphism (SNP) tree using 55 additional *L. monocytogenes* accessions classified the Chilean isolates in lineages I (*n* = 25) and II (*n* = 13). In silico, Multi-locus sequence typing (MLST) differentiated the isolates into 13 sequence types (ST) in which the most common were ST1 (15 isolates) and ST9 (6 isolates) and represented 55% of the isolates. Genomic elements associated with virulence (i.e., LIPI-1, LIPI-3, *inlA*, i*nlB*, *inlC*, *inlG*, *inlH*, *inlD*, *inlE*, *inlK*, *inlF*, and *inlJ*) and stress survival (i.e., stress survival islet 1 and stress survival islet 2) were unevenly distributed among clinical and non-clinical isolates. In addition, one novel *inlA* premature stop codon (PMSC) was detected. Comparative analysis of *L. monocytogenes* circulating in Chile revealed the presence of globally distributed sequence types along with differences among the isolates analyzed at a genomic level specifically associated with virulence and stress survival.

## 1. Introduction

*Listeria monocytogenes* is a foodborne pathogen responsible for listeriosis, which is a severe disease especially in high-risk groups such as the elderly, pregnant women, and newborns [1] in which the case-fatality rate is usually up to 20–30% [2]. Furthermore, *L. monocytogenes* represents a major concern for the food industry due to its ubiquitously distribution in the food production environment and its ability to survive and grow in stress conditions such as acidic environments, high salt concentrations, and low temperatures [3], which are conditions usually found in food preservation barriers.

Subtyping techniques have classified *L. monocytogenes* into four evolutionary lineages and 13 serotypes. Most isolates from clinical cases and food belonging to lineages I and II. These two main lineages contain serotypes 1/2a, 1/2b, and 4b, which represent the most frequently reported serotypes involved in human listeriosis cases and outbreaks [4]. Multi-locus sequence typing (MLST) further subdivided *L. monocytogenes* into 63 phylogenetic groups known as clonal complexes (CC). Some CCs are highly prevalent [5,6] and have been associated with clinical cases worldwide [7].

Pathogenesis of *L. monocytogenes* is associated with their ability to invade, multiply, and survive within different non-phagocytic cells [8]. These characteristics are attributed to the presence of *Listeria* Pathogenicity Island-1 (LIPI-1) and the *inlAB* operon*. Listeria* Pathogenicity Island-1 contains genes that allows *Listeria* to escape from the phagocytic vacuole to replicate in the cytosol and to spread cell-to-cell using actin polymerization [9] and the *inlAB* operon encodes two internalins, which are critical for entry into non-phagocytic cells [10]. In addition, accessory internalin family members have been identified and associated with virulence [11,12,13]. Several studies have shown that subtypes of *L. monocytogenes* differ in their pathogenic potential [14,15,16]. For example, invasion assays in human epithelial cells have shown that some isolates have an attenuated invasion phenotype due to the presence of premature stop codon mutations (PMSC) in *inlA*, which leads to the production of truncated *InlA*. This type of mutations are mostly found in *L. monocytogenes* isolated from foods [17].

Multiple isolates carry genetic elements that could provide them with advantages in food processing environments such as the stress survival islet 1 (SSI-1) and the recently discovered stress survival islet 2 (SSI-2) [18]. These two islets encode genes, which allow *L. monocytogenes* to survive in suboptimal conditions commonly found in food processing environments (i.e., low pH, high salt concentrations, and alkaline and oxidative stress conditions) [18,19].

In Chile, two *L. monocytogenes* outbreaks occurred in 2008 and 2009, which were linked to the consumption of soft cheeses and sausages/meat products, respectively [20]. The pulsed field gel electrophoresis (PFGE) analysis identified two PFGE types, which are the PFGE type 9 for the 2008 outbreak and the PFGE Type 1 for the 2009 outbreak [20]. Furthermore, epidemiological surveillance has shown that sporadic listeriosis cases have slowly increased since 2010 in Chile [21]. Previous reports have described the presence of *L. monocytogenes* in ready-to-eat food (RTE) and raw food products [22,23,24]. In Chile, most isolates belong to serotype 4b and displayed PFGE patterns that suggested the isolates were closely related with human clinical cases [24].

To date, there is a lack of knowledge on the genomic diversity of Chilean *L. monocytogenes* isolates from humans and foods. This study aims to investigate the genomic diversity of *L. monocytogenes* from clinical cases and food in Chile and to put these isolates in a phylogenetic context with regard to isolates from other countries.

## 2. Materials and Methods

### 2.1. Listeria monocytogenes Isolates Used in This Study

A total of 38 isolates obtained from different locations and sources in Chile were selected for whole genome sequencing (WGS) and genomic analysis (Table 1 and Figure S1). These isolates were previously PFGE typed at the Chilean Institute of Public Health. A total of 22 isolates were obtained from clinical samples. In addition, 16 isolates were obtained from food and food-related environments. All isolates were selected to represent different locations in Chile, isolation years, and different PFGE types. Among them, four isolates were of PFGE types 1 and type 9, which represented the PFGE types linked to the listeriosis outbreaks occurred in 2008 and 2009 in Chile.

### 2.2. Genome Sequencing and Annotation

For DNA purification, the DNeasy Blood and Tissue kit (Qiagen, Valencia, CA, USA) was used. The QUBIT fluorimeter (Life Technologies, Carlsbad, CA, USA) was used to quantify the DNA. The Nextera XT DNA library Preparation kit (Illumina, San Diego, CA, USA) was used for library preparation and DNA sequencing was performed on the NextSeq500 (Illumina Inc., San Diego, CA, USA). Sequencing was conducted at the Food and Drug Administration (FDA) Center for Food Safety and Applied Nutrition. *Listeria* genomes were sequenced with a 2 × 150-bp paired-end run. Adapters of the obtained reads were removed and quality trimmed with Trimmomatic (v.0.35) [25]. Reads were analyzed and checked for quality using FastQC (v0.11.4) [26]. The reads were de novo assembled using SPAdes (V3.7.1) [27]. Assemblies were obtained by setting k-mer lengths of 21, 33, 55, and 77 for read lengths between 150 and 300 bp (default settings). Contigs were annotated using a combination of annotation with RAST [28] and automatic annotation with the National Center for Biotechnology Information (NCBI) Prokaryotic Genome Annotation Pipeline (PGAP) [29]. All genomes were deposited at the DDBJ/ENA/GenBank (See Table S1 for accession numbers).

### 2.3. Lineage Determination and Phylogenetic Analysis

Lineage determination was performed using Parsnp from software Harvest suite tools [30]. A core genome alignment of the 38 Chilean isolates along with 55 publicly available sequences (See Table S2) was performed. These isolates of *L. monocytogenes* were used as references for three major lineages (I, II, and III). The phylogenetic relationship of the 38 Chilean isolates was inferred using single nucleotide polymorphisms (SNPs) with Call SNPSs & Infer Phylogeny (CSI) Phylogeny v.1.4, which creates a maximum likelihood tree [31] using *L. monocytogenes* strain EGD-e (NCBI: NC_003210.1) as a reference genome by using default settings (10× or at least 10% of the average depth). A separate phylogenetic analysis was performed for a group of 15 isolates using CSI phylogeny v.1.4 [31], but F2365 (NCBI: AE017262.2) was used as a phylogenetically closer reference.

### 2.4. Subtyping

When silico serotyping was performed with the LisSero v.0.1, [32] a script predicting serogroups for *L. monocytogenes* simulating a PCR of five regions of DNA (lmo118, lmo0737, ORF2110, ORF2829, and prs as an internal amplification control) [32]. This scheme classified isolates on four molecular serogroups known as IIa:1/2a, 3a; IIb:1/2b, 3b, 7; IIc:1/2c, 3c and IVb:4b, 4d, 4e. The sequence type was inferred from WGS data using the program MLST 1.8 from the Center for Genomic Epidemiology [33] and was revised for updated assignments and Clonal complexes by using the Institut Pasteur whole genome MLST database [16]. One novel ST was identified, which was submitted to the Pasteur Institute database to confirm the new assignment. Prophage analysis was performed using PHASTER [34]. The diversity of plasmids was conducted with PlasmidFinder [35] and the presence of antimicrobial resistance genes was screened with ResFinder [36].

### 2.5. Screening of Virulence Genes and Stress-Related Elements

To analyze genes related to virulence and stress survival islets, the BLAST algorithm from NCBI was used [37]. The strain of *Listeria monocytogenes* EGD-e (NCBI: NC_003210.1) was used as a reference for the analysis of SSI-1, LIPI-1, *inlAB* operon, and other internalins (*inlC*, *inlG*, *inlH*, *inlE*, *inlF*, *inlK*, *inlJ*, and *inlD*). For *inlA* characterization, sequences were aligned and screened for non-sense mutations causing premature stop codons or amino acid deletion using the software ClustalO 2.1 [38]. *Listeria monocytogenes* strain F2365 4b was used as a reference for LIPI-3 (NCBI: NC_002973.6) and *L. monocytogenes* strain CDL64 (NCBI: HQ179545.1) was used as a reference for SSI-2. Additionally, VirulenceFinder 1.5 of *Listeria* was used to screen for 81 distinct virulence genes in their database [39].

## 3. Results and Discussion

The present study characterized the genomic diversity of 38 isolates of *L. monocytogenes* from clinical (human) and non-clinical (food and food related environment) samples obtained in different regions of Chile between 2008 and 2011. Genome sizes ranged between 2.89 Mb and 3.11 Mb. The average G + C content was 37.9%. De novo assembly ranged from 12 contigs to 61 contigs with an average mean of length of the contigs or N50 of 454,984 bp (Table S1).

Major findings of this study include: (i) *L. monocytogenes* isolates are mostly represented by CCs distributed worldwide and involved in human infections and outbreaks, (ii) isolates of the PFGE type causing the 2008 to 2009 outbreaks showed genetic relatedness to other worldwide clinical isolates, (iii) clinical and non-clinical *L. monocytogenes* isolates showed distinct virulence and stress survival genetic elements, and (iv) the presence of one novel PMSC mutation in the *inlA* gene along with additional PMSC already reported in other countries in isolates from non-clinical samples.

### 3.1. Listeria monocytogenes Isolates Are Mostly Represented by Clonal Complexes Distributed Worldwide and Involved in Human Infections and Outbreaks

**Figure 1:**
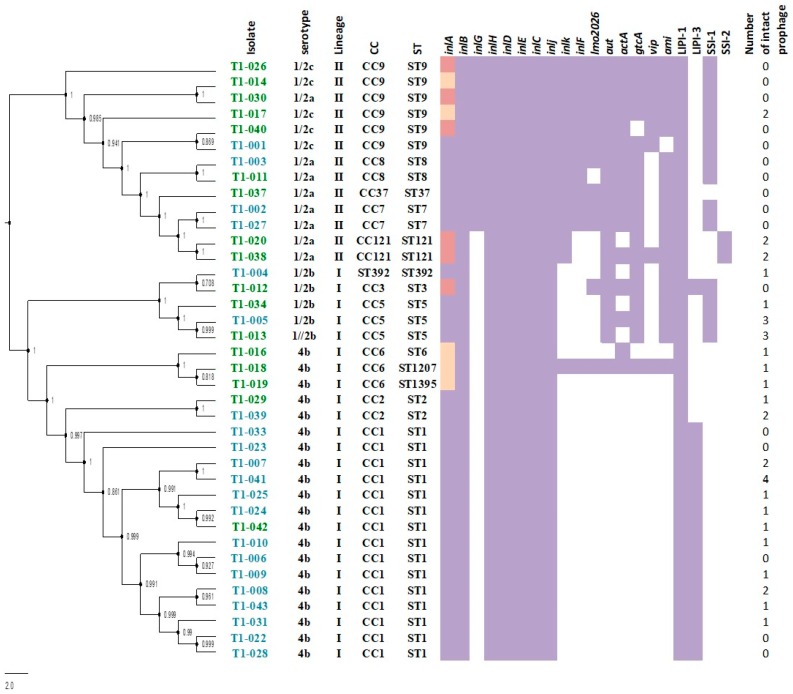
Phylogenetic tree of 38 *L. monocytogenes* isolates from Chile inferred from single nucleotide polymorphisms (SNPs) of whole genome sequencing data and the distribution of their genetic elements associated with virulence and stress survival**.** Isolates from clinical samples are colored in blue and isolates from non-clinical samples are colored in green. Columns on the right of the tree indicate the presence (purple) or absence (white) of genetic elements. Isolates with premature stop codon mutations (PMSCs) in *inlA* are in pink and isolates with 3 aa deletions are yellow. The number of intact prophages predicted with PHAST is added.

A rapid core genome alignment classified the 38 isolates in two lineages (I and II). The majority of clinical isolates grouped in Lineage I (*n* = 25) while the majority of non-clinical isolates grouped in Lineage II (*n* = 13) (Figure S2). Between these two lineages, isolates were classified in four serogroups: serogroup IVb (52.6%), serogroup IIa (21.1%), and serogroups IIb and IIc (13.1% each). Serogroup IVb and IIb belong to Lineage I and serogroup IIa and IIc belong to Lineage II (Table 1 and Figure 1). Strains of serotype 4b (belonging to serogroup IVb) have been responsible for the majority of human listeriosis outbreaks worldwide [40] even though serotype 1/2a and 1/2b have been also involved in outbreaks especially in Europe [41]. In this study, most of the isolates sequenced represented serogroup IVb. Most of the clinical isolates (65%) were of this serogroup while serogroups IIa and IIb were represented by 17% and 13% of the isolates, respectively. One of the clinical isolates was classified as serogroup IIc, which is an uncommon serotype in human clinical cases [42]. Among isolates obtained from non-clinical samples, serogroup IVb were also the most common (33%). This distribution is similar to results previously obtained by Montero et al. (2015) in Chile where serotype 4b was the most prevalent in isolates from food (46%) [24]. Similar findings were reported in China in 2010 [43]. Previous studies have shown that serotypes 1/2b and 4b are the most prevalent in food in Uruguay [44] and serotypes 1/2c and 4b are frequent in food samples from Brazil [45]. Within isolates classified as serogroup IVb, a search for an atypical IVb variant 1 (IVb-1) was conducted [46]. This variant has recently been linked to several outbreaks in the United States [47]. However, none of the isolates were found to represent this variant. While the IVb-1 is considered rare, a recent study identified this variant in isolates of *L. monocytogenes* isolated from frozen prawns in Chile [48].

The MLST analysis differentiated the 38 Chilean isolates into 13 sequence types (STs). A total of 12 STs had been previously reported and were present in the Institut Pasteur whole genome MLST database and one novel ST was assigned (ST1395) (Table 1). Additionally, the 13 STs were grouped in 11 CCs and 1 ST represented a singleton (Figure 1 and Table 1). In Lineage I, isolates were grouped in CC1 (*n* = 15), CC2 (*n* = 2), CC3 (*n* = 1), CC5 (*n* = 3), CC6 (*n* = 3), and one singleton (ST392). In Lineage II, isolates were grouped in CC7 (*n* = 2), CC8 (*n* = 2), CC9 (*n* = 6), CC37 (*n* = 1), and CC121 (*n* = 2). The most common CCs in Lineage I and Lineage II were CC1 (60%) and CC9 (46%), respectively. Most of the CCs identified among Chilean isolates represent the most common CCs worldwide with CC1 and CC9 as the most commonly and widely reported CCs in Europe and South/Central America [5,6]. Importantly, CC1 has also been associated with hyper virulence [16]. In addition, CCs were not equally distributed among isolates from different origins. CC1 was more common on isolates from clinical cases and CC9 in non-clinical isolates. This result is in agreement with a recent retrospective study that analyzed over 6000 strains of *L. monocytogenes* in France [16].

### 3.2. Isolates of the Pulsed Field Gel Electrophoresis Type Causing the 2008–2009 Outbreaks Showed Genetic Relatedness to Other Worldwide Clinical Isolates

The phylogenetic analysis of the core genome alignment identified a group within Lineage I of clinical isolates (T1-006, T1-007, T1-008, T1-009, T1-010, T1-022, T1-025, T1-031, T1-033, T1-041, and T1-043) and one non-clinical isolate (T1-042) that were clustered together (Figures S2 and S3, Table S3). All these isolates belong to the same CC1 and were obtained in different years (2008–2011) and displayed different PFGE types. To gain insights into relatedness between these isolates, an analysis of the whole genome to determine SNP differences among them was conducted using the *L. monocytogenes* strain F2365 as the most closely related reference. SNP differences of these isolates ranged from 17 SNPs to 198 SNPs (Table S3). Within this subgroup, T1-023, T1-024, and T1-028 presented the same PFGE type as the isolates that caused the 2008 outbreak (type 9). However, these three isolates were found to differ between 66 SNPs to 122 SNPs (Table S3) from each other. A previous study using WGS showed a lower diversity among epidemiologically linked isolates (same PFGE type) with SNP differences less than 10 SNPs [49]. A cluster of three non-clinical isolates (T1-016, T1-018, and T1-019), which clustered in CC6 and showed SNP differences that ranged from three SNPs to 12 SNPs was found, which suggests these isolates are highly related even though these isolates were not epidemiologically linked in this study. Prophage analysis on these isolates showed one intact prophage identified in T1-016, T1-018, and T1-019. These two isolates (T1-018 and T1-019) were different by only three SNPs.

### 3.3. Clinical and Non-Clinical L. monocytogenes Isolates Showed Distinct Virulence and Stress Survival Genetic Elements

In this study, the distribution of selected virulence genes and genetic elements related with stress survival were surveyed. Genes encoded in LIPI-1 (*prfA*, *plcA*, *plcB*, *hly*, and *mpl*) and the *inlAB* operon, which encodes internalin A and B, were present in all 38 isolates (Figure 1). A previous study conducted in Chile of *L. monocytogenes* isolated from foods (e.g., raw meat, cheese, and frozen seafood) reported a different distribution of these genomic elements. In the previous study, the LIPI-1 cluster and the *inlAB* operon were found to be associated with a given serotype and food group [24]. However, methodologies and isolates between our study and this previous are different, which may explain the difference in the results. Reports in France and China found these genes in all isolates and both studies used WGS [16,50]. Other internalin family members (*inlC*, *inlJ*, *inlH*, *inlD*, i*nlE*, and *inlJ*) were detected in all isolates (Figure 1). However, *inlG*, *inlF*, and *inlK* were found not evenly distributed among isolates. The genes *inlG* and *inlK* were found in 14 isolates and *inlF* in 12 isolates. Most of these isolates were obtained from non-clinical sources, which are commonly represented by Lineage II. The presence of *inlG* seems to be associated with Lineage II and our result agree with previous studies using PCR that associated the presence of these internalins exclusively with Lineage II [51,52]. Additionally, an analysis looking at 81 distinct genes in the database of VirulenceFinder for *Listeria* identified seven genes distinct to internalins (i.e., *lmo2026*, *aut*, *actA*, *gtcA*, *vip*, and *ami*) that showed diversity in their content (Figure 1). Other virulence markers possibly associated with lineage is LIPI-3, which was exclusively found in 15 isolates of the Lineage I of serotype 4b and one from serotype 1/2b (Figure 1). LIPI-3 has been previously associated with Lineage I, serotype 4b [53,54], and with Lineage III and *Listeria innocua* [55,56]. Conversely, the stress survival associated gene cluster known as SSI-1 was found in both lineages. SSI-1 was found in 37% of isolates and most of these isolates belonged to CC9, CC8, and CC7 of Lineage II and to CC5 and CC3 (both Lineage I). However, SSI-2 was found only in two isolates from CC121 (Lineage II). This is consistent with previous reports that indicate SSI-2 to be only associated with CC121 isolates [18]. The analysis of the plasmids identified that none of the isolates contained a known plasmid. In addition, the only antimicrobial resistance gene identified was the gene *fos*X, which confers resistance to fosfomycin identified in all 38 isolates. The number of prophages detected with PHAST was very diverse and ranged from 0 to 4 intact prophages detected (Figure 1).

### 3.4. Presence of One Novel PMSC Mutation in the inlA Gene Along with Additional PMSC Reported in Other Countries in Isolates from Non-Clinical Samples

The WGS analysis showed that most of the isolates of this study (68%) contained a complete *InlA.* All clinical isolates have a full-length *inlA* gene while 11 isolates from non-clinical samples carried a PMSC mutation (Figure 1). Five isolates harbored a previously reported 9 nucleotide deletion, which was predicted to encode a shorter (797 amino acids) version of InlA. This variant of *inlA* is predicted to be fully functional*.* In vitro invasion assays have shown that these shorter variants have an invasion ability comparable with that of full-length *inlA* isolates [57,58] and also have been reported in isolates from clinical cases [14]. This type of deletion was found in isolates of serotypes 1/2c and 4b and have been found in isolates of serotypes 1/2b in USA [59] as well as serotypes 4c and 1/2a in Canada and Switzerland [60]. Additionally, six PMSCs were detected exclusively in isolates from non-clinical origin, which belongs to serotypes 1/2c (3), 1/2a (2), and 1/2b (1) (Table 2). These mutations were classified into four PMSC types that were previously described, which include one type 19 (resulting in 325 aa protein product), one type 13 (resulting in a 527 aa protein product), two type 6 (resulting in 491 aa protein product), and one type 11 (resulting in a 684 aa protein product) [61,62,63,64]. In addition, the presence of a novel PMSC type was found in one isolate (T1-012), which carried a non-sense mutation at position 821 where one adenine base was deleted. This resulted in a frameshift mutation, which codes for a 277-protein product (Table 2). This truncated protein might result in low virulence in in vitro invasion assays due to the lack of the LPXTG motif, which is involved in anchoring the protein to peptidoglycan in the cell wall [65]. Further studies are essential to confirm this.

## 4. Conclusions

Whole genome sequencing has proven to be a powerful subtyping tool for *L. monocytogenes* especially in reference centers in North America and Europe. This study provides the first characterization at a genomic level using WGS of clinical and non-clinical isolates of *L. monocytogenes* isolated from Chile. Our results show the presence of isolates from Chile that represent clonal groups associated with listeriosis worldwide, which supports the global distribution of key human diseases associated with *L. monocytogenes* clonal groups. This study is the first of this type in South America, so further efforts are necessary in order to implement WGS in routine surveillance in South America.

## Figures and Tables

**Table 1:** Metadata and molecular characterization of *L. monocytogenes* isolates used in this study.

Isolates	Origin ^1^	Source	Isolation Date	Geographic Location ^2^	Pulse Type ^3^	Serogroup ^4^	Sequence Type ^4^	Clonal Complex ^4^
T1-001	Clinical	Amniotic fluid	2009	Santiago	167	IIc	ST9	CC9
T1-002	Clinical	Blood	2010	Santiago	3	IIa	ST7	CC7
T1-003	Clinical	Cerebrospinal fluid	2011	Los Lagos	114	IIa	ST8	CC8
T1-004	Clinical	Cerebrospinal fluid	2011	Santiago	260	IIb	ST392	-
T1-005	Clinical	Cerebrospinal fluid	2010	Valparaiso	19	IIb	ST5	CC5
T1-006	Clinical	Blood	2010	Aysén	48	IVb	ST1	CC1
T1-007	Clinical	Blood	2010	Santiago	197	IVb	ST1	CC1
T1-008	Clinical	Cerebrospinal fluid	2011	Araucanía	235	IVb	ST1	CC1
T1-009	Clinical	Amniotic fluid	2011	Santiago	252	IVb	ST1	CC1
T1-010	Clinical	Blood	2011	O’Higgins	264	IVb	ST1	CC1
T1-011	Food	Ham	2010	Santiago	167	IIa	ST8	CC8
T1-012	Food	Sausage	2010	Los Lagos	147	IIb	ST3	CC3
T1-013	Food	Ice cream	2010	Santiago	212	IIb	ST5	CC5
T1-014	Food	Sausage	2010	Los Lagos	210	IIc	ST9	CC9
T1-016	Food	Ham	2010	Santiago	211	IVb	ST1207	CC6
T1-017	Food	Ham	2010	Santiago	99	IIc	ST9	CC9
T1-018	Food	Ice cream	2010	Santiago	156	IVb	ST1395 ^7^	CC6
T1-019	Environment	Food plant	N/A	Bío-Bío	N/A	IVb	ST6	CC6
T1-020	Environment	Food plant	N/A	Bío-Bío	N/A	IIa	ST121	CC121
T1-022	Clinical	Blood	2011	Valparaiso	256	IVb	ST1	CC1
T1-023	Clinical	Blood	2008	Santiago	9 ^5^	IVb	ST1	CC1
T1-024	Clinical	Blood	2008	O’Higgins	9 ^5^	IVb	ST1	CC1
T1-025	Clinical	Blood	2010	Santiago	99	IVb	ST1	CC1
T1-026	Food	Sausage	2010	Los Lagos	2	IIc	ST9	CC9
T1-027	Clinical	Blood	2010	Santiago	46	IIa	ST7	CC7
T1-028	Clinical	Blood	2008	Bío-Bío	9 ^5^	IVb	ST1	CC1
T1-029	Food	Pork pate	2010	Araucanía	126	IVb	ST2	CC2
T1-030	Food	Sausage	2009	Bío-Bío	1 ^6^	IIa	ST9	CC9
T1-031	Clinical	Human	2010	O’Higgins	20	IVb	ST1	CC1
T1-033	Clinical	Blood	2010	Araucanía	133	IVb	ST1	CC1
T1-034	Food	Ice cream	2010	Santiago	64	IIb	ST5	CC5
T1-037	Clinical	Peritoneal fluid	2011	Bío-Bío	137	IIa	ST37	CC37
T1-038	Food	Ham	2010	Santiago	53	IIa	ST121	CC121
T1-039	Clinical	Cerebrospinal fluid	2010	Santiago	209	IVb	ST2	CC2
T1-040	Food	Beef	2009	Bío-Bío	1 ^6^	IIc	ST9	CC9
T1-041	Clinical	Blood	2011	Bío-Bío	245	IVb	ST1	CC1
T1-042	Food	Cheese	2009	Santiago	9 ^5^	IVb	ST1	CC1
T1-043	Clinical	Blood	2010	Santiago	58	IVb	ST1	CC1

N/A: Not available. ^1^ All clinical cases were obtained from humans. ^2^ For details on geographic origin within Chile, see Figure S1. ^3^ Pulsed Field Gel Electrophoresis (PFGE) were typed at the Chilean Institute of Public Health. ^4^ Identified in this study. ^5^ PFGE type was involved in outbreak 2008. ^6^ PFGE type was involved in outbreak 2009. ^7^ Novel sequence type.

**Table 2:** Length of *Inl*A among Chilean *L. monocytogenes.*

Number of Isolates	*InlA* Length (aa)	Mutation Type (PMSC)	Nucleotide Mutation Position	Functional	Reference
27	800	-	-	yes	Glaser et al., 2001
5	797	NA	NA	yes	Chen et al., 2011
1	684	11	2054 (G to A)	no	Rousseaux et al., 2002
1	527	13	1579 (Ato T)	no	Handa-Miya et al., 2007
2	491	6	1474 (Cto T)	no	Olier et al., 2002
1	325	19	976 (G to T)	no	Gelbicova et al., 2015
1	277	Novel	821 (deletion A)	no	In this study

## Supplementary Materials

The following are available online at http://www.mdpi.com/2073-4425/9/8/396/s1. Figure S1. Location and distribution of isolates used in this study. (a) Map of Chile divided by regions and red dots represent the locations where the isolates were obtained. (b) Circular representation of the percent of isolates sequenced that were obtained from the different regions within Chile. Figure S2. Maximum likelihood phylogenetic tree based on SNPs of core genome of Chilean *L. monocytogenes* using *55 L. monocytogenes* sequences as references. Phylogenetic tree representing the two lineages identified in this study, ID of the isolates from clinical samples are colored in blue and ID of the isolates from non-clinical samples are colored in green. Figure S3. *L. monocytogenes* phylogeny based on single nucleotide polymorphism (SNP). Phylogenetic tree of the 15 isolates that clustered together that were obtained from clinical samples, mostly of the CC1. Isolates with their ID in blue were obtained from human clinical samples and the one in green from non-clinical samples. The bootstrap was added to the clades. Table S1. Sequencing statistics of 38 isolates of *L. monocytogenes* obtained from clinical and non-clinical samples sequenced in this study*.* Table S2. *L. monocytogenes* used as references in the core genome alignment for lineage determination. Table S3. Pairwise distance matrix of SNP number differences for 15 Chilean isolates of *L. monocytogenes* clustered in CC1.
