# Supplementary material for: Genomic Diversity of Listeria monocytogenes Isolated from Clinical and Non-Clinical Samples in Chile
Source: Genes (Basel). 2018 Aug 2;9(8):396. doi: 10.3390/genes9080396 (PMC6115834; doi:10.3390/genes9080396)
Supplement: Supplementary file 1 [file genes-09-00396-s001.zip › FigureS1.pdf]

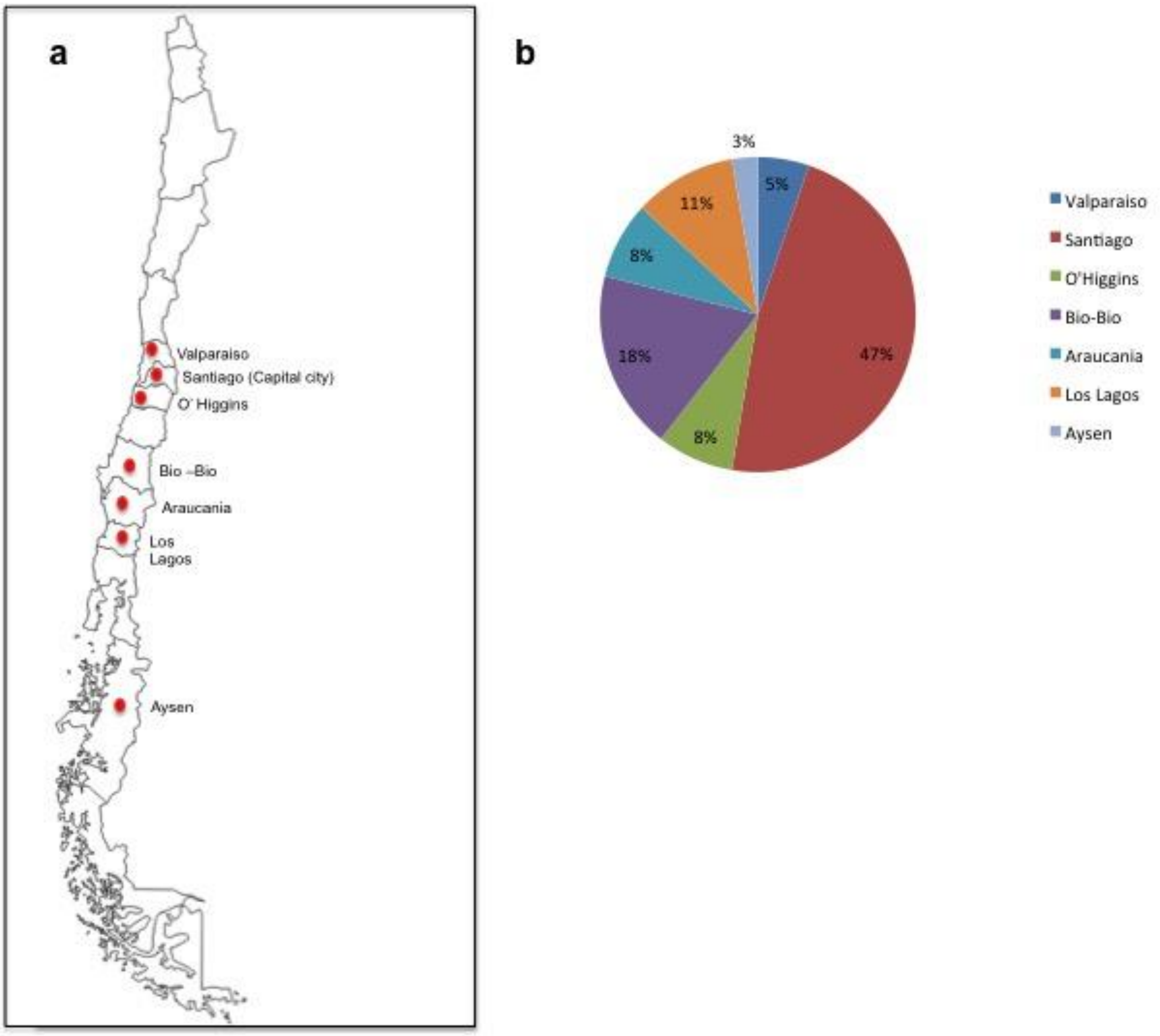

Figure S1. Location and distribution of isolates used in this study. (a) Map of Chile divided by regions and red dots represent the locations where the isolates were obtained. (b) Circular representation of the percent of isolates sequenced that were obtained from the different regions within Chile
