# Supplementary material for: Genomic Diversity of Listeria monocytogenes Isolated from Clinical and Non-Clinical Samples in Chile
Source: Genes (Basel). 2018 Aug 2;9(8):396. doi: 10.3390/genes9080396 (PMC6115834; doi:10.3390/genes9080396)
Supplement: Supplementary file 1 [file genes-09-00396-s001.zip › FigureS2.pdf]

genes

MDPI

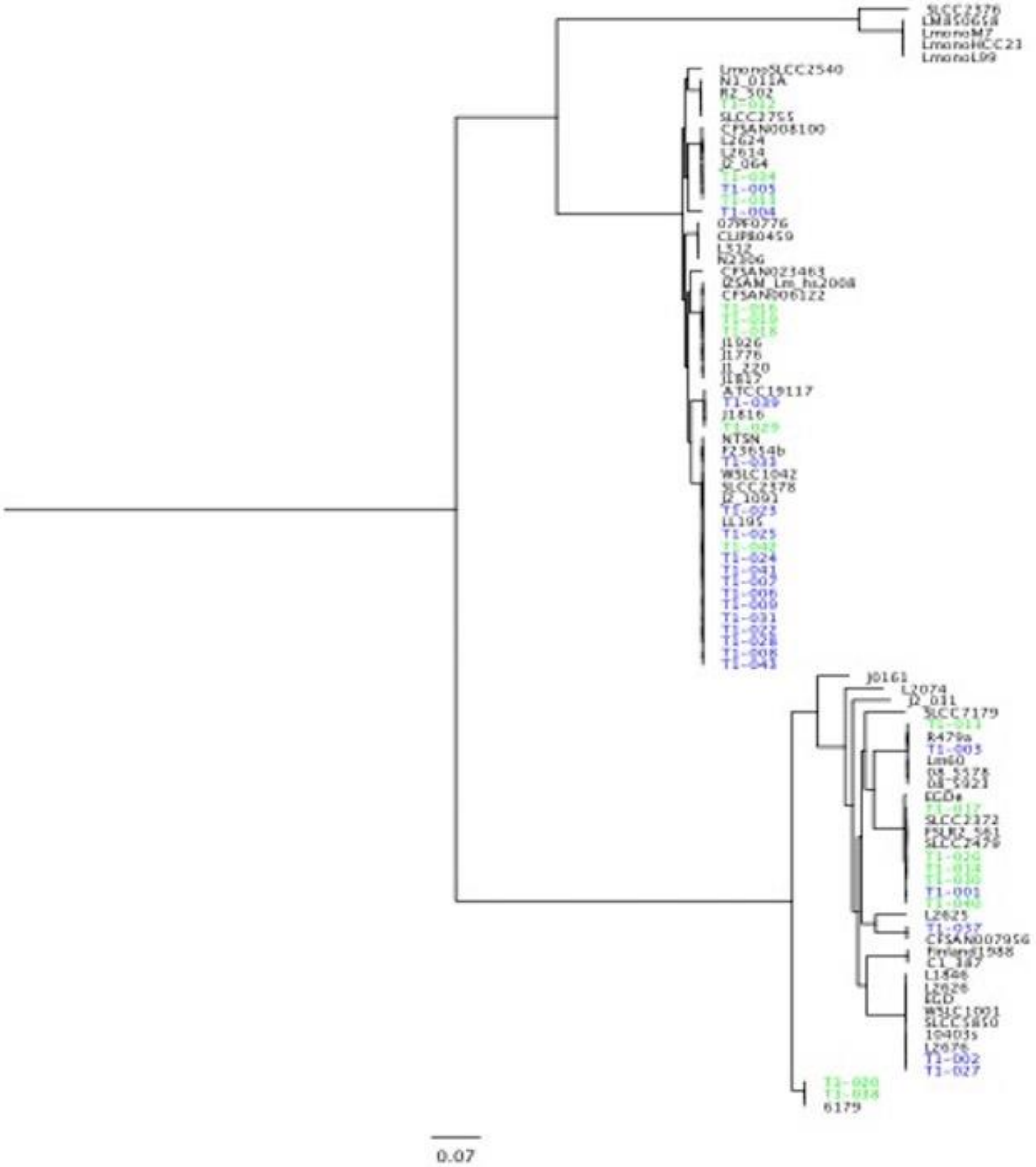

Figure S2. Maximum likelihood phylogenetic tree based on SNPs of core genome of Chilean *L. monocytogenes* using *55 L. monocytogenes* sequences as references. Phylogenetic tree representing the two lineages identified in this study, ID of the isolates from clinical samples are colored in blue and ID of the isolates from non-clinical samples are colored in green.
