# Supplementary material for: Genomic Diversity of Listeria monocytogenes Isolated from Clinical and Non-Clinical Samples in Chile
Source: Genes (Basel). 2018 Aug 2;9(8):396. doi: 10.3390/genes9080396 (PMC6115834; doi:10.3390/genes9080396)
Supplement: Supplementary file 1 [file genes-09-00396-s001.zip › FigureS3.pdf]

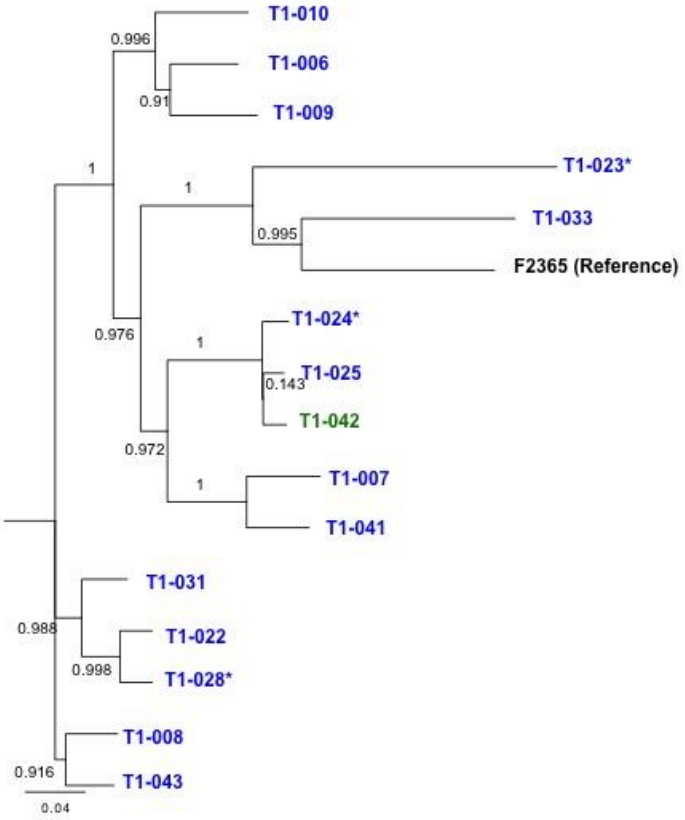

Figure S3. *Listeria monocytogenes* phylogeny based on single nucleotide polymorphism (SNP). Phylogenetic tree of the 15 isolates that clustered together that were obtained from clinical samples, mostly of the CC1. Isolates with their ID in blue were obtained from human clinical samples and the one in green from non-clinical samples. The bootstrap was added to the clades.
