# Supplementary material for: Genomic Diversity of Listeria monocytogenes Isolated from Clinical and Non-Clinical Samples in Chile
Source: Genes (Basel). 2018 Aug 2;9(8):396. doi: 10.3390/genes9080396 (PMC6115834; doi:10.3390/genes9080396)
Supplement: Supplementary file 1 [file genes-09-00396-s001.zip › TableS1.pdf]

Table S1. Sequencing statistics of 38 isolates of *L. monocytogenes* obtained from clinical and non-clinical samples and sequenced in this study.

Isolate	GC content	Number of CDS	Genome size (Mbp)	Contigs	N50	Genbank accession no.
T1-001	37.9	3071	3.05	20	429,602	PVUO00000000
T1-002	37.9	2917	2.91	20	477,812	PVUP00000000
T1-003	37.8	2918	2.92	12	579,709	PVUQ00000000
T1-004	37.9	2964	2.97	21	501,516	PVUR00000000
T1-005	37.9	3107	3.10	26	516,487	PVUS00000000
T1-006	37.8	2882	2.91	22	477,225	PVUT00000000
T1-007	37.8	3084	3.07	61	237,958	PVUU00000000
T1-008	37.9	2935	2.95	24	481,271	PVUV00000000
T1-009	37.9	2985	2.98	27	381,601	PVUW00000000
T1-010	37.8	2987	2.99	21	600,094	PVUX00000000
T1-011	37.9	2921	2.92	14	438,610	PVUY00000000
T1-012	37.9	2989	3.03	16	511,024	PVUZ00000000
T1-013	37.9	3107	3.10	30	516,391	PVVA00000000
T1-014	37.9	3049	3.04	19	448,549	PVVB00000000
T1-016	37.9	2893	2.92	24	514,953	PVVC00000000
T1-017	37.9	3073	3.02	27	434,210	PVVD00000000
T1-018	37.8	2891	2.92	24	514,953	PVVE00000000
T1-019	37.8	2893	2.92	21	514,952	PVVF00000000
T1-020	37.9	3002	3.01	22	522,776	PVVG00000000
T1-022	37.8	2819	2.87	23	521,710	PVVH00000000
T1-023	37.9	2857	2.90	22	517,387	PVVI00000000
T1-024	37.9	2874	2.91	24	300,180	PVVJ00000000
T1-025	37.8	2875	2.91	22	295,002	PVVK00000000
T1-026	37.8	2998	2.99	29	477,703	PVVL00000000
T1-027	37.8	2915	2.91	14	502,192	PVVM00000000
T1-028	37.9	2818	2.87	24	521,505	PVVN00000000
T1-029	37.9	2952	2.99	19	331,006	PVVO00000000
T1-030	37.9	2919	2.96	25	341,851	PVVP00000000
T1-031	37.9	2939	2.95	29	381,659	PVVQ00000000
T1-033	37.9	2917	3.10	23	520,563	PVVR00000000
T1-034	37.8	3087	3.09	36	436,191	PVVS00000000
T1-037	37.9	2811	2.84	19	450,121	PVVT00000000
T1-038	37.8	3053	3.06	18	563,450	PVVU00000000
T1-039	38.0	3052	3.04	42	254,269	PVVV00000000
T1-040	37.8	3008	3.10	19	477,705	PVVW00000000
T1-041	37.9	3100	3.09	35	480,494	PVVX00000000
T1-042	37.9	2879	2.91	24	295,002	PVVY00000000
T1-043	37.9	2884	2.91	21	521,711	PVVZ00000000
