# Supplementary material for: Genomic Diversity of Listeria monocytogenes Isolated from Clinical and Non-Clinical Samples in Chile
Source: Genes (Basel). 2018 Aug 2;9(8):396. doi: 10.3390/genes9080396 (PMC6115834; doi:10.3390/genes9080396)
Supplement: Supplementary file 1 [file genes-09-00396-s001.zip › TableS2.pdf]

genes

MDPI

Table S2. *L. monocytogenes* used as references in the core genome alignment for lineage determination.

Isolate name	Country	Source	Collection Date	Serotype	Lineage	Clonal complex (CC)	Sequence-type (ST)	Genbank Accession no.
07PF0776	USA	Human	2011	4b	I	CC4	ST4	CP003414.1
08-5578	Canada	Human	2008	1/2a	II	CC121	ST121	CP001602.2
08-5923	Canada	Human	2008	1/2a	II	CC121	ST121	CP001604.1
6179	Ireland	Cheese	2014	1/2a	II	CC121	ST121	HG813249.1
10403S	USA	Human	1964	1/2a	II	CC7	ST85	CP002002.1
ATCC 19117	N/A	Sheep	N/D	4d	I	CC2	ST2	FR733643
C1-387	USA	Food	1999	1/2a	II	-	-	CP006591.1
CFSAN006122	USA	Cheese	2013	N/A	I	CC6	ST6	CP007600.1
CFSAN007956	USA	N/A	N/D	N/A	II	CC37	ST37	CP011397.1
CFSAN008100	USA	Green chile	N/A	N/A	I	CC5	ST5	CP011398
CFSAN023463	USA	Peaches	2014	N/A	-	N/D	N/D	CP012021.1
CLIP80459	N/A	N/A	2013	4b	I	-	-	FM242711.1
EGD-e	UK	Guinea pig	1926	1/2a	II	CC9	ST35	AL591824.1
EGD	UK	Guinea pig	1926	1/2a	II	CC7	ST12	HG421741.1
F23654b	USA	Cheese	1985	4b	I	CC1	ST1	AE017262.2
Finland1998	Finland	Butter	1998	3a	II	CC155	ST155	CP002004.1
FSLR2-561	USA	Human	N/D	1/2c	II	CC9	ST122	CP002003.1
HCC23	USA	Animal	N/A	4a	III	-	-	CP001175.1
IZSAM_Lm_hs2008	Italy	Human	2009	4b	I	-	-	CP010346
J0 161	USA	Human	2000	1/2a	II	CC11	ST11	CP002001.1
J1 220	USA	Human	N/A	4b	I	-	-	CP006046.2
J1 776	USA	Human	2002	4b	I	CC6	ST6	CP006598.1
J1 816	USA	Food	2002	4b	I	-	-	CP006047.2
J1 817	USA	Environment	2002	4b	I	CC6	ST6	CP006599.1
J1 926	USA	Food	2002	4b	I	CC6	ST6	CP006600.1
J2 031	USA	Human	1996	1/2a	II	CC415	ST394	CP006593.1
J2 064	USA	Animal	1989	1/2b	I	-	-	CP006592.1
J1 108	Canada	Coleslaw	1981	4b	I	-	-	CP006596.1
L1846	USA	Human	2009	1/2b	I	-	-	CP007688

L2074	USA	Human	2010	1/2a	II	-	-	CP007689
L2624	USA	Human	2011	1/2b	I	-	-	CP007686.1
L2625	USA	Human	2011	1/2a	II		ST29	CP007687.1
L2626	USA	Cantaloupe	2011	1/2a	II	CC7	ST561	CP007684.1
L2676	USA	Human	2011	1/2a	II	CC7	ST7	CP007685
L312	N/A	Cheese	N/A	4b	I	CC4	ST4	FR733642.2
L99	Netherlands	Cheese	195	4a	III	-	ST-201	FM211688
LL195	Switzerland	Human	2013	4b	I	-	-	HF558398.1
Lm60	Switzerland	Human	2006	1/2a	II		ST551	CP009258.1
Lm850658	Australia	Sheep	1985	4b	-	-	-	CP009242.1
M7	China	Milk	N/A	4a	III	-	-	CP002816.1
N1-011A	USA	Environment	N/A	1/2b	I	-	-	CP006597.1
N2306	Switzerland	Human	2014	4b	I	CC4	ST4	CP011004.1
NTSN	China	Sheep	2011	-	-	-	-	CP009897.1
R2 502	USA	Food	1994	1/2b	I	CC3	ST3	CP006594.1
R479a	Dinamarca	Salmon	1996	1/2a	II	CC8	ST8	HG813247.1
SLCC2372	Reino unido	Human	1935	1/2c	II	CC9	ST122	FR733648.1
SLCC 2376	N/A	Chicken	N/A	4c	III	CC131	ST71	FR733651.1
SLCC 2378	N/A	Chicken	N/A	4e	I	CC1	ST73	FR733644.1
SLCC 2479	N/A	N/A	1966	3c	II	CC9	ST9	FR733649.1
SLCC 2540	USA	Human	1956	3b	I	-	-	FR733645.1
SLCC 2755	N/A	Chinchilla	1927	1/2b	I	CC3	ST66	FR733646.1
SLCC 5850	UK	Rabbit	1924	1/2a	II	CC7	ST12	FR733647.1
SLCC 7179	Austria	Cheese	1986	3a	II	CC14	ST91	FR733650.1
WSLC1001	Germany	N/A	N/A	1/2a	II	-	-	CP007160.1
WSLC1042	Germany	N/A	N/A	4b	I	-	-	CP007210.1

N/A not available
